# Response of Medical Cannabis (*Cannabis sativa* L.) Genotypes to K Supply Under Long Photoperiod

**DOI:** 10.3389/fpls.2019.01369

**Published:** 2019-11-18

**Authors:** Avia Saloner, Mollie M. Sacks, Nirit Bernstein

**Affiliations:** ^1^Institute of Soil, Water and Environmental Sciences, Volcani Center, Rishon LeZion, Israel; ^2^The Faculty of Agriculture, Food and Environment, The Hebrew University of Jerusalem, Rehovot, Israel; ^3^Shaham, Extension Service, Ministry of Agriculture, Rishon LeZion, Israel

**Keywords:** cannabis, cultivation, development, fertilizer, nutrition, potassium, vegetative, genotype

## Abstract

Potassium is involved in regulation of multiple developmental, physiological, and metabolic processes in plants, including photosynthesis and water relations. We lack information about the response of medical cannabis to mineral nutrition in general, and K in particular, which is required for development of high-grade standardized production for the medical cannabis industry. The present study investigated the involvement of K nutrition in morphological development, the plant ionome, photosynthesis and gas-exchange, water relations, water use efficiency, and K use efficiency, comparatively for two genotypes of medical cannabis, under a long photoperiod. The plants were exposed to five levels of K (15, 60, 100, 175, and 240 ppm K). Growth response to K inputs varied between genotypes, revealing genetic differences within the *Cannabis sativa* species to mineral nutrition. Fifteen ppm of K was insufficient for optimal growth and function in both genotypes and elicited visual deficiency symptoms. Two hundred and forty ppm K proved excessive and damaging to development of the genotype Royal Medic, while in Desert Queen it stimulated rather than restricted shoot and root development. The differences between the genotypes in the response to K nutrition were accompanied by some variability in uptake, transport, and accumulation of nutrients. For example, higher levels of K transport from root to the shoot were apparent in Desert Queen. However, overall trends of accumulation were similar for the two genotypes demonstrating competition for uptake between K and Ca and Mg, and no effect on N and P uptake except in the K-deficiency range. The extent of accumulation was higher in the leaves > roots > stem for N, and roots > leaves > stem for P. Surprisingly, most micronutrients (Zn, Mn, Fe, Cu, Cl) tended to accumulate in the root, suggesting a compartmentation strategy for temporary storage, or for prevention of access concentrations at the shoot tissues. The sensitivity of net-photosynthetic rate, gas exchange, and water use efficiency to K supply differed as well between genotypes. The results suggest that growth reduction under the deficient supply of 15 ppm K was mostly due to impact of K availability on water relations of the tissue and transpiration in Royal Medic, and water relations and carbon fixation in Desert Queen.

## Introduction

Cannabis (*Cannabis sativa L*.) has been cultivated by mankind from antiquity, for medical (Zuardi, 2006; Clarke and Merlin, 2013) and recreational use (Small, 2017), and as a source for seed oil and fibers (Leizer et al., 2000; Lash, 2010). Among the plant’s acknowledged medical properties are anti-inflammatory potential and easing symptoms of numerous medical conditions including post-traumatic stress disorder, multiple sclerosis, cancer, Crohn’s disease, pain, and chemotherapy (Naftali et al., 2013; Greer et al., 2014; Cascio et al., 2017). The diverse medical potential is predicated on the complex chemical profile, comprising hundreds of secondary metabolites including cannabinoids, terpenes, and flavonoids.

Environmental conditions such as mineral nutrients (Bernstein et al., 2011) and water availability (Wang et al., 2018) affect plant development and function, including synthesis of secondary metabolites in medicinal plants (Eliašová et al., 2004; Figueiredo et al., 2008; Nascimento and Fett-Neto, 2010; Gorelick and Bernstein, 2014). Well documented is the strong connection between potassium (K), one of the principle nutrient elements required by higher plants, and plant development and function (Pettigrew and Meredith, 1997; Bernstein et al., 2011; Grzebisz et al., 2013; Reviewed by Prajapati and Modi, 2012; Tsialtas et al., 2016). K is involved in multiple physiological and metabolic processes, including photosynthesis, transport of assimilates, protein synthesis, enzyme activation, stomata regulation, and osmoregulation, and it is therefore not surprising that it is a key player in regulation of plant development processes (Reviewed by Szczerba et al., 2009; Prajapati and Modi, 2012; Wang et al., 2013; Wang and Wu, 2017). Moreover, K is also known as a ‘quality element’ (Usherwood, 1985). By its effect on the secondary metabolite profile, it improves factors which are of relevance for yield quality such as color, taste and aroma (Reviwed by Usherwood, 1985; Prajapati and Modi, 2012), and hence stands as one of the main targets for study in the medical cannabis research. We lack information about K effects on plant development and function in medical cannabis. Such information is vital for developing optimal fertigation practices to support excelled plant growth and development during the vegetative growth phase, as well as for optimal reproductive development and secondary metabolism during the short day phase.

Legal restrictions during the last decades prevented progress in academic research involving the cannabis plant. This has resulted in meager science-based information about cannabis, which is peculiar considering that it is one of our most ancient crops with a rich history of usage by humanity. We lack basic information about plant developmental and physiological responses to key environmental factors including mineral nutrition, and this hinders efforts to develop high-grade standardized production for the booming medical cannabis market.

*C. sativa* is a “short day” plant, which under long photoperiod undergoes continuous vegetative growth, with inflorescence initiation and development occurring following transition to a short photoperiod. The intensity of growth and the developmental pattern under long photoperiod in cannabis plants, together with the duration of this growth phase, determines plant architecture and size at the onset of the transition to the short photoperiod. The vegetative growth phase is hence a major player in determining the size, architecture, and to a large extent also the spatial pattern of inflorescences distribution in the mature medical cannabis plants, factors which affect yield quantity as well as the potential for standardization of the chemical quality. Understanding and regulating development at the long photoperiod phase is therefore fundamental for excelled quantity and quality production in medical cannabis. Potassium, being a key nutrient for growth and developmental processes should be studied for its effects during this phase. The present study therefore focused on the developmental and physiological responses of medical cannabis at the long photoperiod growth phase to K nutrition.

The little knowledge available about cannabis growth is mainly from research with hemp; a vigorous, tall and woody fiber-type of *C. sativa*. The data collected over the years about industrial hemp indicate that its growth and yield can be greatly affected by fertilization (Bócsa et al., 1997; Ivonyi et al., 1997; Vera et al., 2010; Finnan and Burke, 2013), and that the concentration of cannabinoids such as CBN and CBD can be affected by stress, nutrient deficiency, and other environmental parameters (Haney and Kutscheid, 1973). In spite of their importance, these results for hemp can shed only partial light on medical cannabis physiology and development considering the differences in plant development, genetics and growing practices. The little agro-scientific knowledge available for medical cannabis suggests some interesting correlations between soil pH, nutritional elements, and cannabinoids. These correlations, were determined for seeded plants from an “Afgan origin,” grown in 11 different soil types (Coffman and Gentner, 1975) as part of an attempt to identify cultivation sites of confiscated illegal plant parts. Recently we have demonstrated gradients of cannabinoids and inorganic nutrients along the medical cannabis plants, with an interplay between plant organs and organic and inorganic constituents (Bernstein et al., 2019a). Enhanced mineral nutrition by supplementation of NPK, P, or humic acids, affects specific cannabinoid concentrations in a compound and organ specific manner (Bernstein et al., 2019b), demonstrating the potential of specific mineral nutrients for regulation of growth and the chemical profile.

In the present study we studied comparatively the response of two cultivars of medical cannabis to increasing concentrations of K inputs. The following hypotheses were testes: 1. K supply induces developmental and morphological changes in medical cannabis. 2. The K induced changes in growth and development are associated with changes to the plant ionome and the physiological state of the tissue. To test these hypotheses, we studied effects of K supply ranging from 15 to 240 ppm K in the irrigation solution on mineral uptake and accumulation in the plant organs (leaves, stems, and roots), morphological, and physiological characteristics. The concentration range evaluated was selected to encompass deficiency–sufficiency- and oversupply concentrations, based on the limited information available from growers and a preliminary study conducted by us. Apart from the contribution to understanding of cannabis physiology, the information obtained is instrumental also for development of optimal fertigation regime for excelled quantity and quality product for the agro-hi-tech medical industry.

## Materials and Methods

### Plant Material and Growing Conditions

Two medical cannabis (*C. sativa L.*) cultivars (genotypes), “Royal Medic” (RM) and “Desert Queen” (DQ) (Teva Adir LTD, Israel), which are approved for commercial medical use in Israel, were used as a model system in this study. They are both of indica characteristics, and were selected to represent two distinct chemotypes: “RM” contain similar concentrations of THC and CBD (about 5%), and “DQ” is a high THC cultivar. Plants were propagated from cuttings of a single mother plant in coconut fiber plugs (Jiffy international AS, Kristiansand, Norway). Rooted cuttings were planted in 3 L plastic pots in perlite 2-1-2 (Agrekal, Habonim Israel), and the irrigation treatments were initiated following 7 days adjustment with only distilled water irrigation. The plants were then divided into five increasing treatments of K supply; 15, 60, 100, 175, and 240 ppm, and grown for 30 days under 18/6 h light/dark photoperiod using Metal Halide bulbs (400 μmol*m^−2^*s^−1^, Solis Tek Inc, Carson, California) in a controlled environment growing room. Temperatures in the growing room were 26 and 25˚C day/night, and the relative humidity were 54% and 50%, respectively. Irrigation was supplied *via* a 1 L h^−1^ discharge-regulated drippers (Netafim, Tel-aviv, Israel), 1 dripper per pot. The volume of irrigation in each irrigation pulse was 250–650 ml/pot/day, set to allow 30% of drainage. Fertilizers were supplied by fertigation, i.e., dissolved in the irrigation solution at each irrigation. The irrigation solution contained 14.82 mM N-NO_3_^−^, 1.62 mM N-NH_4_^+^, 1.9 mM P-PO_4_^−2^, 2.99 mM Ca^+2^, 1.45 mM Mg^+2^, 1.04 mM Na^+^, 0.37 mM Cl^−^, 0.03 mM Fe^+2^, 0.02 mM Mn^+2^, 0.005 mM Zn^+2^, and increasing concentrations of K: 0.38, 1.53, 2.56, 4.48, and 6.14 mM K^+^. K was supplemented as K_2_SO_4_ because in preliminary experiments sulfur uptake into the medical cannabis plants was found to be affected less then accumulation of Cl or NO_3_ when K was supplemented as KCl or KNO_3_. The micronutrients were supplied chelated with EDTA, other than Fe that was chelated with EDDHSA. The experiment was arranged in a complete randomized design. All measurements were conducted for five replicated plants and results are presented as average ± standard error (S.E.).

### Inorganic Mineral Analysis

For the analyses of inorganic mineral contents in the plant, the plants were destructively harvested five times throughout the experiment duration; 0, 7, 14, 21, and 29 days after the initiation of the K fertigation treatments. At each sampling event, the sectioned shoots were rinsed twice with distilled water and blotted dry, the leaves were carefully excised from the stem at the point of attachment to the node, and fresh and dry biomass were measured with a Precisa 40SM-200A balance (Zurich, Switzerland). Dry weights were determined following drying at 64˚C for 48 h and the dry tissue was ground to a powder.

The plant samples were analyzed for concentrations of N, P, K, Ca, Mg, S, Fe, Mn, Zn, Cu, Cl, and Na. Three different procedures were applied for extraction of the various inorganic mineral elements from the grounded plant tissue. For the analysis of S, Ca, Mg, Fe, Zn, Cu, and Mn, the ground tissue was digested with HNO_3_ (65%) and HClO_4_ (70%), and the elements (except S) were analyzed with an atomic absorption spectrophotometer, AAnalyst 400 AA Spectrometer (PerkinElmer, Massachusetts, USA). For the analysis of N, P, K, and Na, the dry tissue was digested with H_2_SO_4_ (98%) and H_2_O_2_ (70%–72%). Na and K were analyzed by flame photometer (410 Flame Photometer Range, Sherwood Scientific Limited, The Paddocks, UK), and N, P, and S were analyzed by an autoanalyzer (Lachat Instruments, Milwaukee, WI, USA). For the analyses of Cl, dried plant samples were extracted with a dilute acid solution containing 0.1 N HNO_3_. Cl was measured by potentiometric titration (PCLM3 Jenway, Bibby Scientific Ltd, T/As Jenway, Dunmow, UK). Mineral analyses of irrigation and drainage solutions were performed as described for the plant extraction and digestion solutions.

### Physiological Parameters

The plants were sampled for physiological parameters analyses 31 days after the rooted cuttings were planted in the experimental pots, 24 days after the initiation of the fertigation treatments.

**Determination of osmotic potential.** For osmotic potential measurements, the youngest mature fan leaf on the main stem of the plant, located at the fourth node from the plant’s top was carefully removed, washed twice in distilled water, and blotted dry. The 3 smallest leaflets were cut from the leaf and inserted into a 1.7 micro-test-tube. The tube were then frozen in liquid nitrogen and stored at −20°C for further analyses. The frozen tissue was crushed inside the tubes with a glass rod, the bottom of the tubes was pin-pricked and the tubes, set inside another 1.5 ml tube, centrifuged for 5 min in a refrigerated centrifuge (Sigma Laboratory Centrifuges, Germany) at 4°C at 6,000 rpm. Fifty microliters of the fluids collected in the lower micro test tube were used for measurement of osmotic potential using a cryoscopic microosmometer Osmomat 3000 (Gonotec, Berlin, Germany) by measuring the freezing point of 50 µl of sap. Results are presented in mOsm kg^−1^ H_2_O^−1^. Five replicated leaves from five replicated plants for each cultivar were analyzed.

**Determination of membrane leakage.** Ion leakage from the leaf tissue, an indicator of membrane injury under stress (Lu et al., 2008), was measured as previously described (Shoresh et al., 2011) with minor modifications. The youngest mature fan leaf on the main stem of the plant, located at the fourth node from the plant’s top was carefully removed, washed twice in distilled water and blotted dry. A 40-mm segment located at the central of the middle leaflet was used for the analysis. The sampled leaf section was rapidly placed in a 50 ml test-tube containing 30 ml of distilled water and shaken for 24 h. The electric conductivity of the soaking solution containing the leaf was measured using a conductivity meter Cyberscan CON 1500 (Eutech Instruments Europe B.V., Nijkerk, Netherlands). Then, the samples were autoclaved for 30 min to destroy cells and cause 100% leakage. The autoclaved samples were allowed to cool down at room temperature for 45 min and then shaken for an additional 1 h. The electric conductivity of the solution was measured. Ion leakage from the plant tissue was calculated as percent (%) of the electric conductivity value before autoclaving to its value post autoclaving. Results from five replicated leaves from five replicated plants for each cultivar were averaged.

**Determination of chlorophyll and carotenoids content.** For chlorophyll and carotenoid analysis, the youngest mature fan leaf on the main stem of the plant, located at the fourth node from the plant’s top was carefully removed, washed twice in distilled water, and blotted dry. Five discs, 0.6 cm in diameter, were cut from the second largest leaflet, placed in 0.8 ml 80% (v/v) ethanol, and were frozen for further analysis. After partial thawing at room temperature, the samples were heated to 100°C for 30 min. The soluble boiled extract was collected in 2 ml micro test tubes. The remaining tissue was extracted again in 0.5 ml 80% (v/v) ethanol for 15 min at 100°C and the combined extract was mixed by vortex. Next, 0.4 ml of extract was transferred to 5 ml 80% (v/v) acetone, and absorbance at 663, 646, and 470 nm was measured by Genesys 10 UV Scanning spectrophotometer (Thermo Scientific, Waltham, Massachusetts). Calculation of chlorophyll a and b and carotenoids was done according to Lichtenthaler and Welburn (1983).

**Determination of relative water content.** For relative water content analysis, the second youngest mature fan leaf on the main stem of the plant, located at the fifth node from the plant’s top was carefully removed, and weighed with a Precisa 40SM-200A balance (Zurich, Switzerland). The leaf was then placed in a 50 ml rube that was previously filled with distilled water. The tubes were placed for 24 h at room temperature and then the leaves were blotted dry and weighed again. Dry weight of the leaves was obtained following desiccation at 64˚C for 48 h. Relative water content was calculated following (Bernstein et al., 2010). The analyses was conducted for five replicated leaves from five replicated plants, for each cultivar.

**Plant architecture and development.** Plant height, stem diameter, and the number of nodes on the main stem were measured five times throughout the experiment duration; 0, 8, 15, 22, and 28 days after the initiation of the fertigation treatments. Plant height and branch length were measured with a ruler from the base of the plant to the top of the central branch. Stem diameter was measured with an Electronic digital caliper YT-7201 (Signet tools international co., LTD., Shengang Distric, Taiwan) at the location 5 cm from the plant base. The measurements were conducted on five replicated plants per treatment, for each cultivar.

**Photosynthesis and transpiration rate, stomatal conductance, intercellular CO**_2_ **concentration, and water use efficiency.** Net photosynthesis rate, intercellular CO_2_ concentration, transpiration rate, stomatal conductance, and water use efficiency quantification were measured on the youngest mature fan leaf on the main stem of the plant, located at the fourth node from the plant’s top, with a Licor 6400 XT system (LI-COR, Lincoln, NE, USA). The leaves were exposed to a light intensity of 400 PPFD and a CO_2_ concentration of 400 ppm while leaf temperature was kept at 25°C and relative humidity was between 40% and 55%. Water use efficiency was calculated from the net photosynthesis and stomatal conductance results. The measurements were conducted on five replicated plants per treatment, for each cultivar.

### Plant Biomass

Distribution of plant biomass between the various vegetative shoot organs, i.e., leaves and stems, was evaluate by destructive sampling five times throughout the experiment duration; 0, 7, 14, 21, and 29 days after the initiation of the fertigation treatments. At the last destructive sampling, on day 29, the roots were gently rinsed three times in distilled water and blotted dry, and fresh weights were measured. Dry weights were measured following desiccation in 64˚C for 48 h. Potassium use efficiency (KUE) was calculated as the total dry weight of the plant on day 29 divided by the amount (g/plant) of K supplied to the plant throughout the experiment duration. Presented results are averages ± SE for five replicated plants.

### Statistical Analyses

The data were subjected to two-way ANOVA followed by Tukey’s HSD test. Comparison of relevant means was conducted using Fisher’s least significant difference (LSD) at 5% level of significance. The analysis was performed with the Jump software (Jump package, version 9 (SAS 2015, Cary, NC, USA).

## Results

### Plant Growth and Development

**Figure 1:**
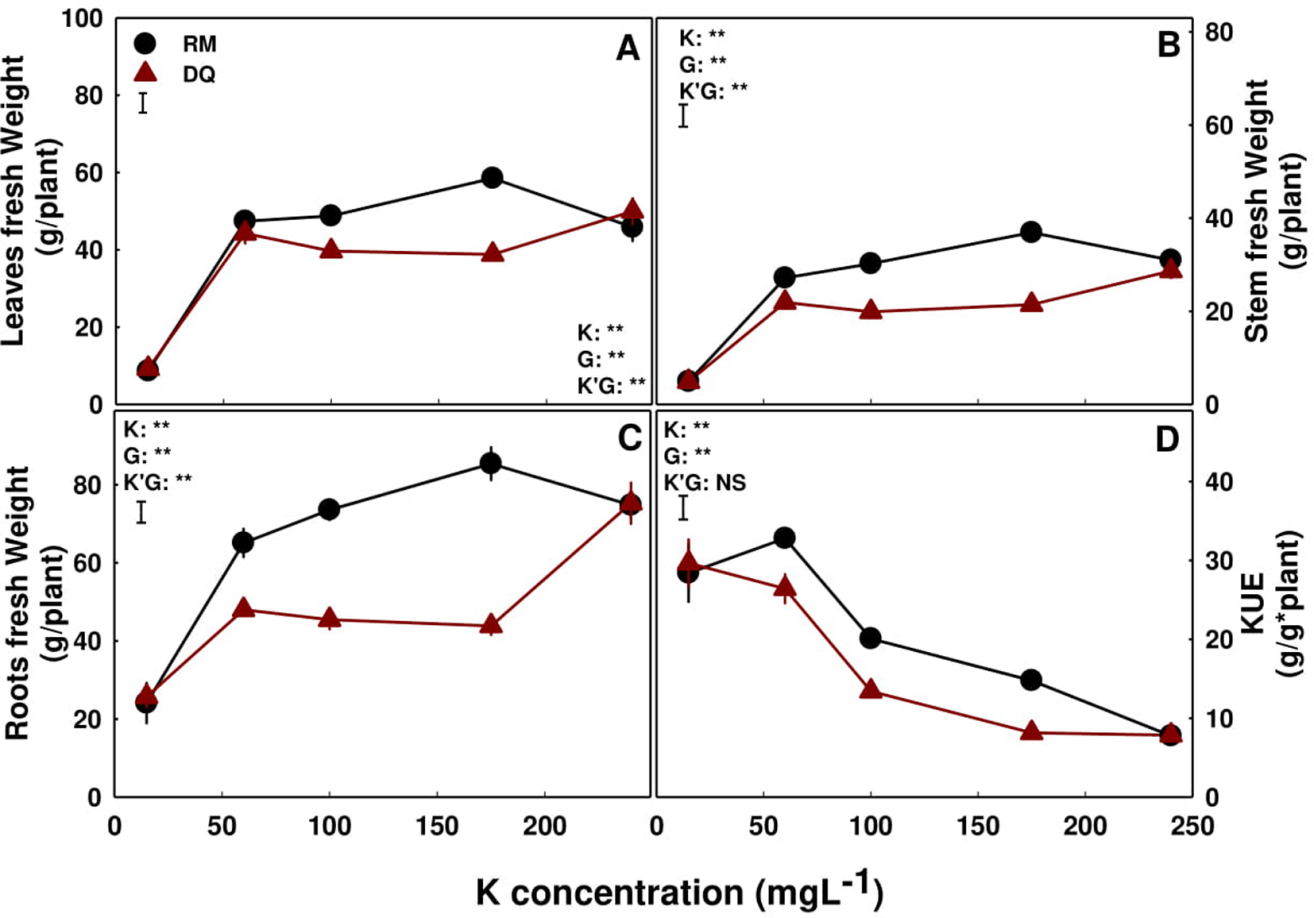
Effect of K nutrition on shoot and root biomass in cannabis plants. Fresh weights of leaves **(A)**, stem **(B)**, and roots **(C)**, and K use efficiency (KUE) **(D)** of two medical cannabis cultivars, Royal Medic (RM) and Desert Queen (DQ). Presented data are averages ± SE (n = 5). Results of two-way ANOVA indicated as **P < 0.05, *F*-test; NS, not significant *P >* 0.05, *F*-test. The bars represent the LSD between means at *P* ≤ 0.05. In the ANOVA results K is potassium, G is genotype, and K'G represents the interaction between K and G.

Shoot and root growth of “RM” plants increased with the elevation of K supply. Biomass of leaves, stems, and roots increasing with the increase in K concentration, up to 175 ppm K, and decreasing with further increase in concentration (**Figures 1A–C**) hence presenting 15 ppm as a sub-optimal concentration and 175 ppm as an optimal K concentration. “DQ” plants are less sensitive to K application, and the shoot and roots demonstrated an unusual yet similar growth response to increasing concentrations of K. The biomass of all three organs was lowest under 15 ppm K supply (a deficient supply), unchanged at the range of 60–175 ppm K, and surprisingly significantly increased with further increase in concentration to 240 ppm K (**Figures 1A–C**). Response patterns of dry biomass accumulations to K supply were similar to the fresh biomass response (data not shown). For both cultivars, KUE was similar for the 15 and 60 ppm K treatments, and decreased with further increase in K supply (**Figure 1D**).

**Figure 2:**
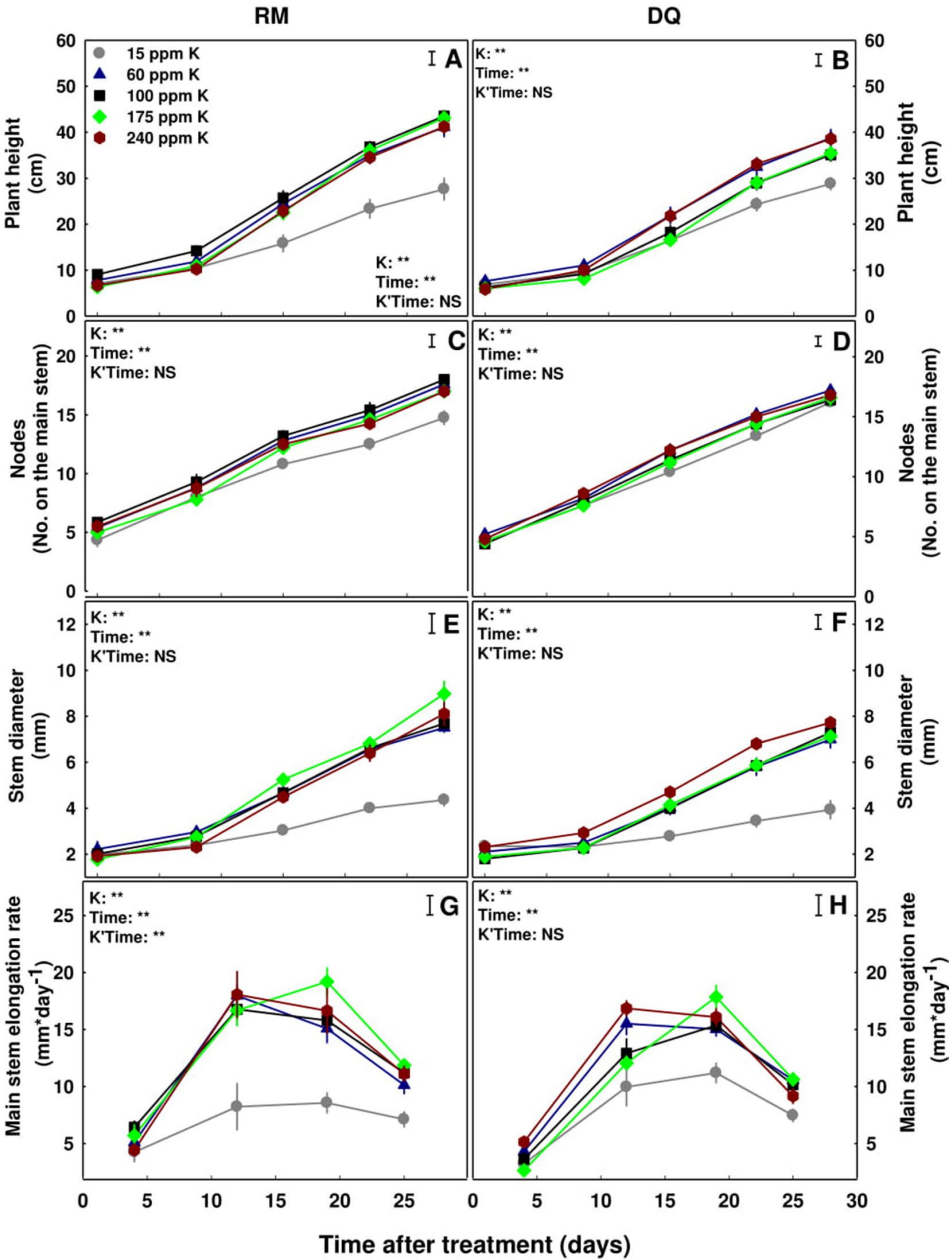
Effect of K nutrition on development of cannabis plants. Plant height **(A**, **B)**, number of nodes on the main stem **(C**, **D)**, stem diameter **(E**, **F)**, and main stem elongation rate **(G**, **H)** of two medical cannabis cultivars, RM and DQ. Presented data are averages ± SE (n = 5). Results of two-way ANOVA indicated as **P < 0.05, *F*-test; NS, not significant *P* > 0.05, *F*-test. The bars represent the LSD between means at P ≤ 0.05. In the ANOVA results, K Time represents the interaction between K and time.

All the morphological parameters measured: plant height, number of nodes on the main stem, stem diameter, and main stem elongation rate, showed a developmental delay in the plants that received 15 ppm K, compared with plants that received higher amounts of K (**Figures 2A–H**) presenting as well 15 ppm as a sub-optimal concentration for both varieties. The two varieties demonstrated a similar response to the elevation of K supply. The average rate of plant elongation in the 15 ppm K treatment was 33% lower in RM and 28% lower in DQ (8.17 and 7.82 mm*day^−1^, respectively), compared to the remaining treatments (**Figures 2G, H**). The average rate of stem thickening in the 15 ppm K treatment was 61% lower in RM and 70% lower in DQ (85 and 56 μm*day^−1^, respectively), compared to the higher K supply treatments (**Figures 2E, F**). The average rate of node formation on the main stem was only 13% lower in RM and 3% lower in DQ in the 15 ppm K treatment (0.37 and 0.41 nodes*day^−1^, respectively), compared to the remaining treatments (**Figures 2C, D**). These developmental delays under the restricted K supply of 15 ppm K had a considerable effect on plant growth, resulting in shorter plants (**Figures 2A, B**). The growth restriction in RM and the stimulation in DQ under high K supply (240 ppm), which is statistically significant for leaves and root biomass of the entire plant (**Figure 1**), is not significant for most of the morphological and growth parameters of the main stem (**Figure 2**). This may present lower sensitivity of the main stem compared to the side branches to over-supply of K, and/or present that side branch development is more sensitive to high supply of K than initiation of new branches (new internodes).

### Macronutrient Concentration

**Figure 3:**
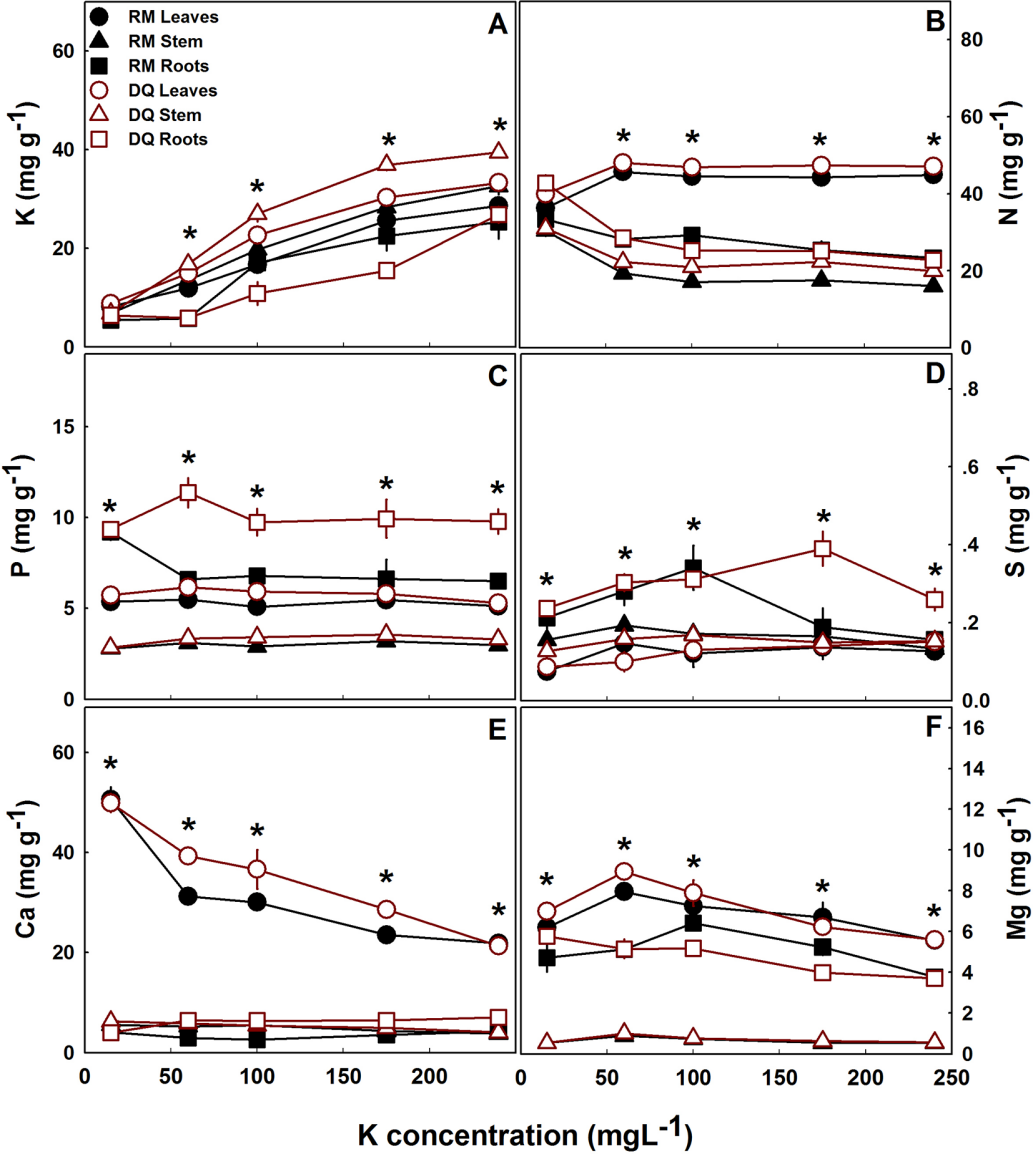
Effect of K supply on macronutrient concentrations in leaves, stem and roots of two medical cannabis cultivars, RM and DQ. K **(A)**, N **(B)**, P **(C)**, S **(D)**, Ca **(E)**, and Mg **(F)**. Presented data are averages ± SE (n = 5). Asterisk above the bars represent significant differences between treatments by Tukey HSD test at α = 0.05.

The concentration of K supplied caused a variety of responses related to the ability of the plants to take up and accumulate nutrients, demonstrating organ but not cultivar specificity, with both cultivars revealing similar responses. In both cultivars, in all plant parts, K concentration increased significantly with increased K supply (**Figure 3A**). The concentration of the two major cation nutrients, Ca and Mg, tended to decrease with increased K supply, demonstrating competition for uptake (**Figures 3E, F**). While K concentration was highest in the stem, Ca and Mg concentration was very low in the stem, and higher in the leaves. Nitrogen and P concentrations in the plant organs were not affected by the level of K supplied, except in the 15 ppm K treatment. Furthermore, the extent of accumulation differed between organs and was higher in the leaves > roots > stem for N, and roots > leaves > stem for P (**Figures 3B, C**). Sulfur concentration in the plant organs was low, with preferred accumulation in the root and highest accumulation under 60–175 ppm K supply (**Figure 3D**).

### Micronutrients and Na

**Figure 4:**
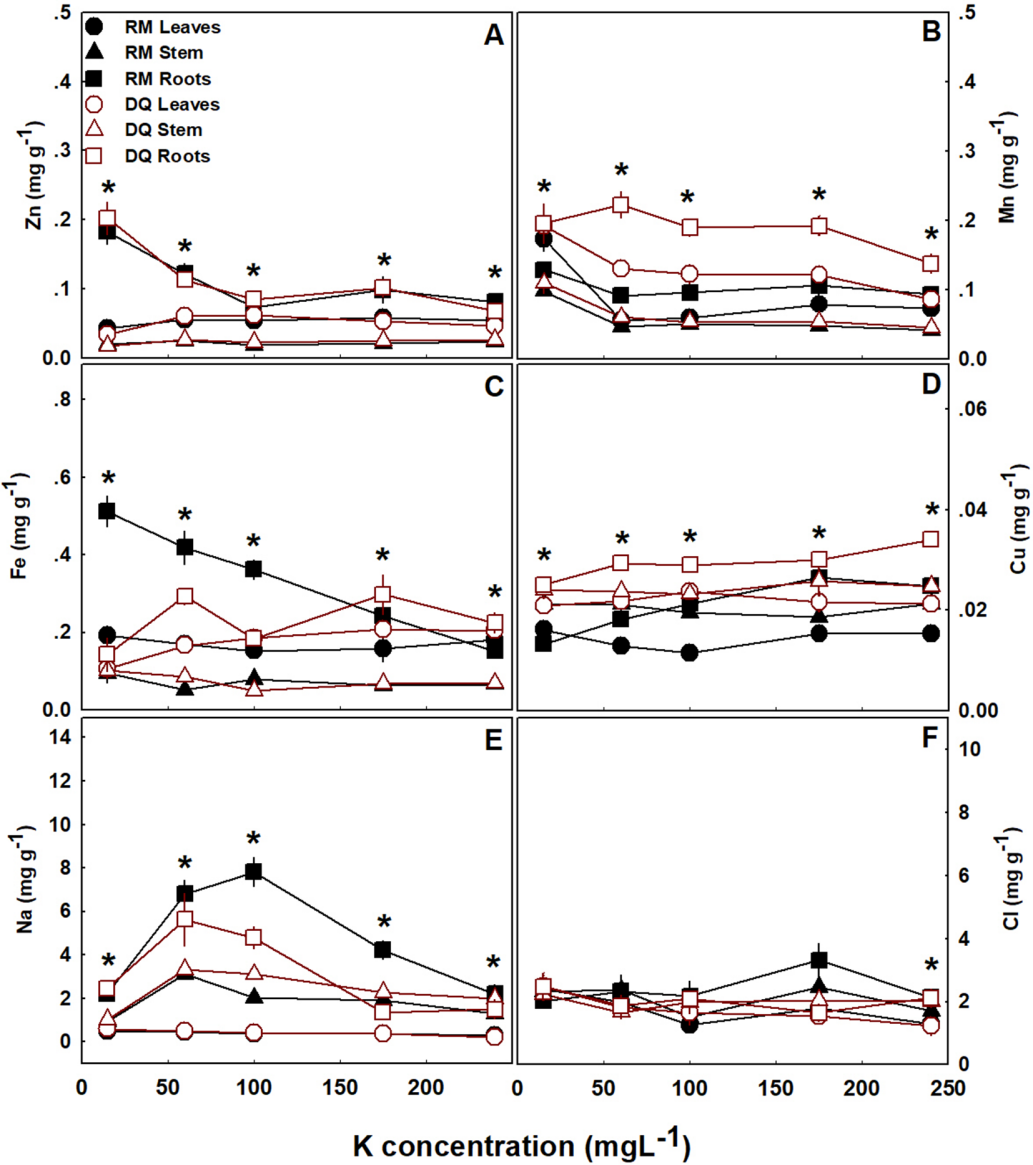
Effect of K supply on micronutrient and Na concentrations in leaves, stem, and roots of two medical cannabis cultivars, RM and DQ. Zn **(A)**, Mn **(B)**, Fe **(C)**, Cu **(D)**, Na **(E)**, and Cl **(F)**. Presented data are averages ± SE (n = 5). Asterisks represent significant differences between organs at each K level, by Tukey HSD test at α = 0.05.

Unlike the considerable effects on macronutrients, K supply had but little effect on micronutrient accumulation in the shoot. Micronutrient concentration in the leaves and stems were generally unaffected by K supply, except Mn which built up to higher concentrations at the 15 ppm K treatment (**Figures 4A–F**). In the roots, concentrations of Zn, Fe, and Mn usually decreased with the increase in K supply; Cu and Na concentration followed maximum curves with highest accumulation under 100–175 ppm K supply (**Figures 4A–E**); and Cl concentrations which were very low were unaffected by K supply (**Figure 4F**). The concentrations of all micronutrients were higher in the roots compared to the shoot. In the shoot, the concentration of Na, Cl, and Cu were generally higher in the stem, compared to the leaves, while an opposite trend was found for the remaining micronutrients (**Figures 4A–F**). The two tested cultivars responded similarly to K fertigation in terms of micronutrient and macronutrient accumulation.

### Gas Exchange and Photosynthesis

**Figure 5:**
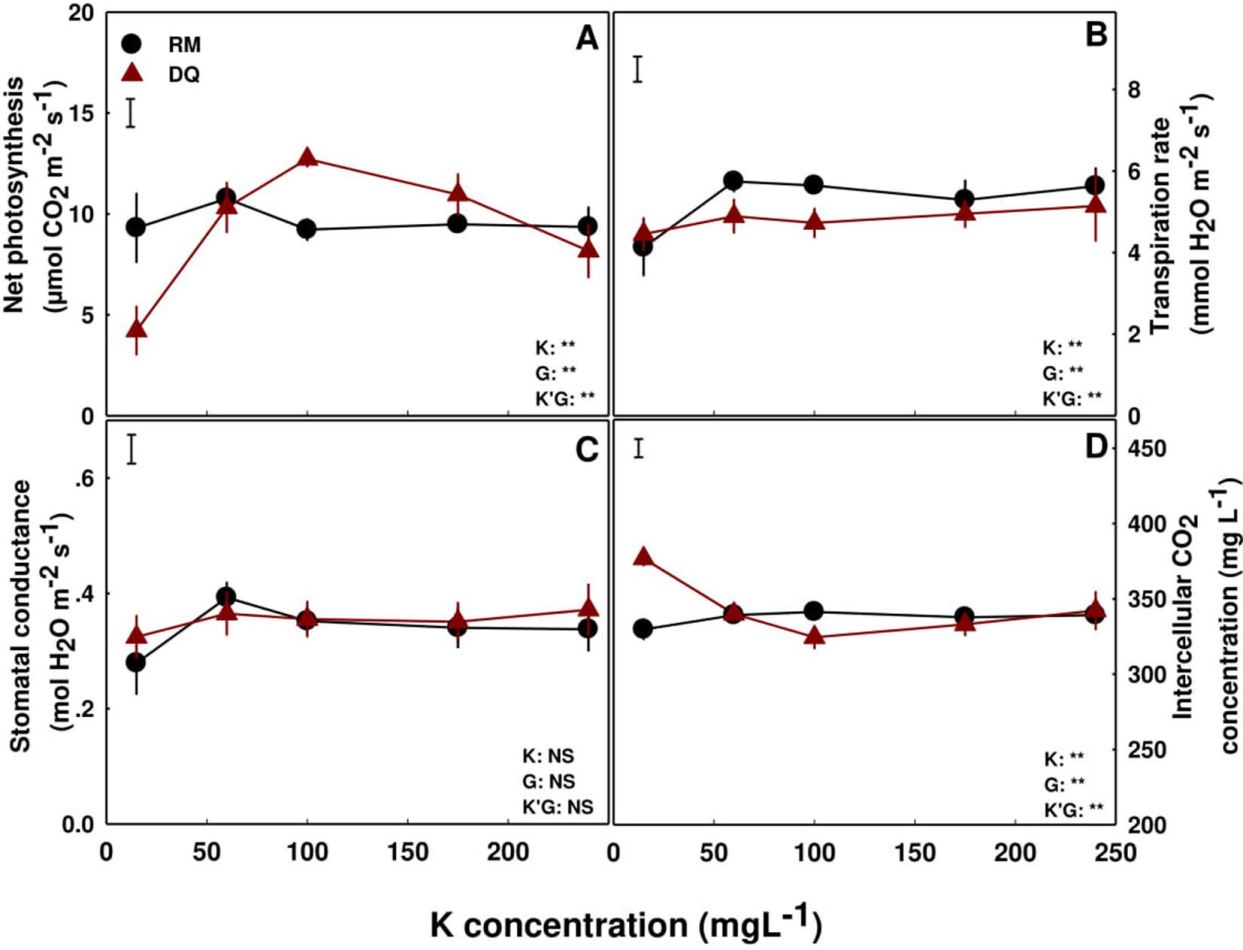
Effect of K supply on gas exchange in cannabis leaves. Net photosynthesis rate **(A)**, transpiration rate **(B)**, stomatal conductance **(C)**, and intercellular CO_2_ concentration **(D)** for two medical cannabis cultivars, RM and DQ. Presented data are averages ± SE (n = 5). Results of two-way ANOVA indicated as **P < 0.05, *F*-test; NS, not significant *P* > 0.05, *F*-test. The bars represent the LSD between means at P ≤ 0.05. In the ANOVA results K'G represents the interaction between K and genotype.

The sensitivity of net photosynthetic rate and gas exchange parameters to K supply differed in the two cultivars. In DQ, net photosynthetic rate increased with the increase in K supply, up to a maximum at the 100 ppm K treatment, and declined with further increase in K (**Figure 5A**) presenting 100 ppm as the optimal concentration for photosynthesis in this cultivar. In contrast, the intercellular CO_2_ concentration was higher at the 15 ppm K treatment than in all other treatments, and the transpiration rate and stomatal conductance was not affected by K supply (**Figures 5B–D**). RM responded differently, with transpiration rate and stomatal conductance lower at the 15 ppm K treatment compared to all other treatments, and net photosynthetic rate and intercellular CO_2_ concentration were not affected by the K supply treatments (**Figures 5A–D**).

### Water Relations and Photosynthetic Pigments

**Figure 6:**
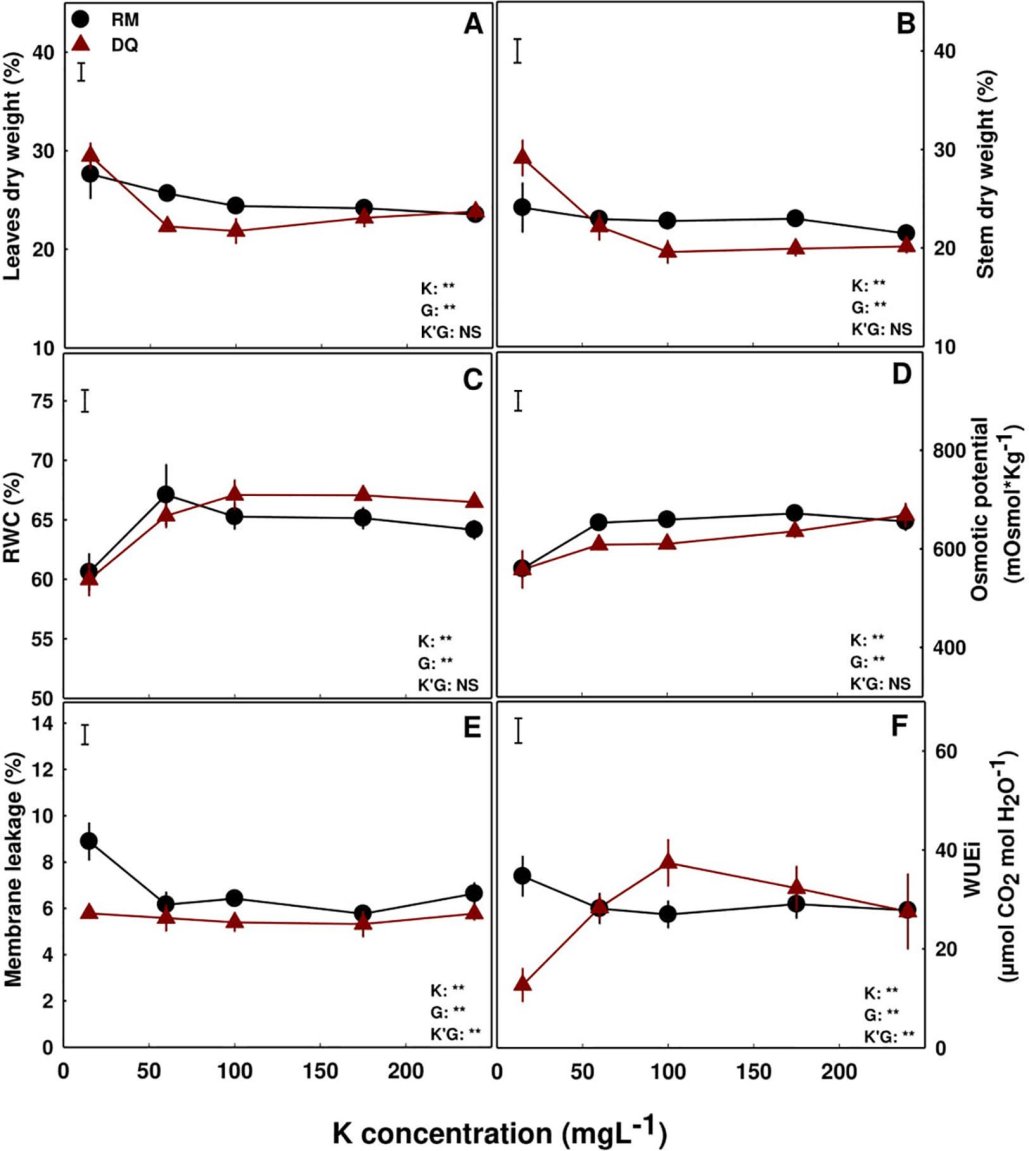
Physiological characteristics of medical cannabis plants. %DW of leaves **(A)** and stems **(B)**, relative water content (RWC) **(C)**, osmotic potential **(D)**, membrane leakage **(E)** and intrinsic water use efficiency (WUEi) **(F)**, of two medical cannabis cultivars, RM and DQ. Presented data are averages ± SE (n = 5). Results of two-way ANOVA indicated as **P < 0.05, F-test; NS, not significant *P* > 0.05, F-test. In the ANOVA results K'G represents the interaction between K and genotype.

The effect of K supply on the percentage of dry weight (%DW) in the plant tissues differed between the two varieties. In DQ, %DW of the leaves and stems was higher at the 15 ppm K treatment compared to all other treatments (**Figures 6A, B**), while in RM, %DW of the stem was not affected by the treatments but in the leaves it was higher under 15 and 60 ppm K supply, compared to other K supply treatments. Osmotic potential and relative water content of the leaf were lower under 15 ppm K supply, compared to all other treatments, in both varieties (**Figures 6C, D**). In DQ the osmotic potential increased with additional K supply throughout the concentration range tested, while in RM it stabilized under a lower K supply (60 ppm K; **Figure 6D**). Membrane leakage analyses demonstrated a higher sensitivity of RM tissues to K deficiencies (15 ppm K) compare to DQ, and a lack of response in both varieties to higher K supply (Fig 6E). In RM water use efficiency was not affected by K supply, while in DQ it was significantly lower in the 15 ppm K treatment, compared to all other higher K treatments (**Figure 6F**).

**Figure 7:**
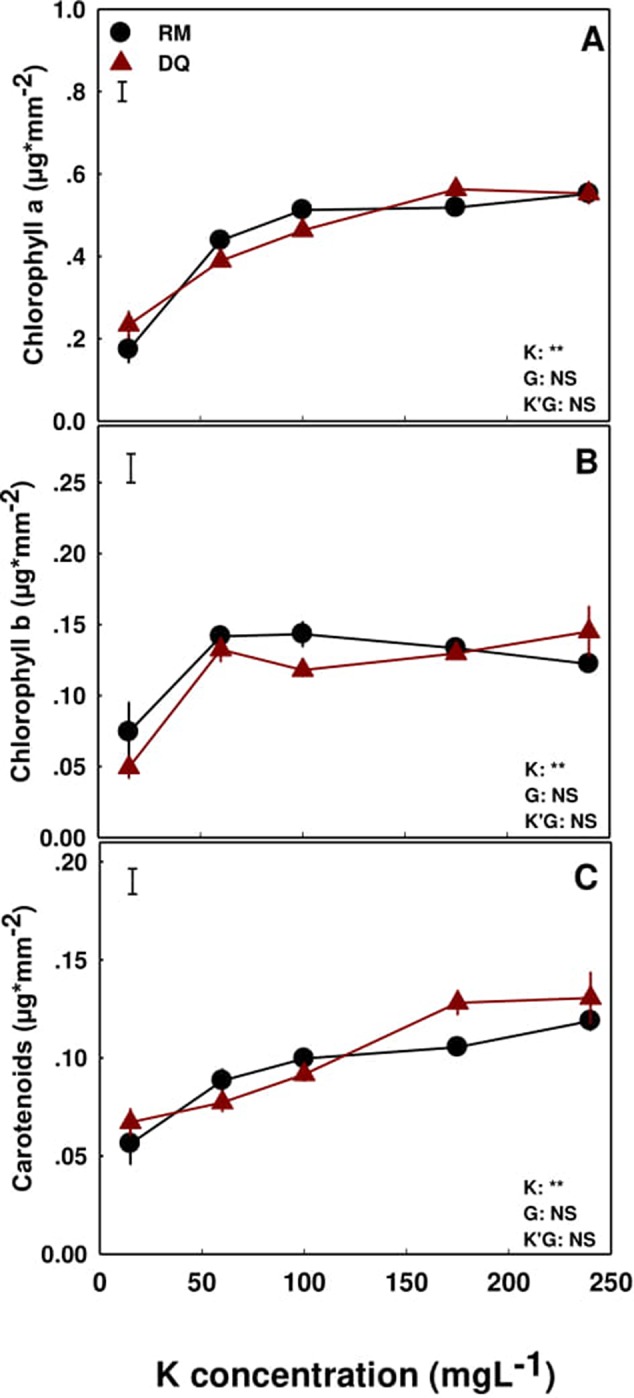
Effect of K application on the concentration of photosynthetic pigments in two medical cannabis cultivars, RM and DQ. Chlorophyll a **(A)**, chlorophyll b **(B)**, and carotenoids **(C)**. Presented data are averages ± SE (n = 5). Results of two-way ANOVA indicated as **P < 0.05, *F*-test; NS, not significant *P* > 0.05, *F*-test. The bars represent the LSD between means at P ≤ 0.05. In the ANOVA results K’G represents the interaction between K and genotype.

Concentrations of chlorophyll a and carotenoids in the foliage increased with elevation of K supply (**Figures 7A, C**), while chlorophyll b was significantly lower at the 15 ppm K treatment, compared to the remaining treatments, in both varieties (**Figure 7B**).

### Irrigation and Leachate Solutions

**Figure 8:**
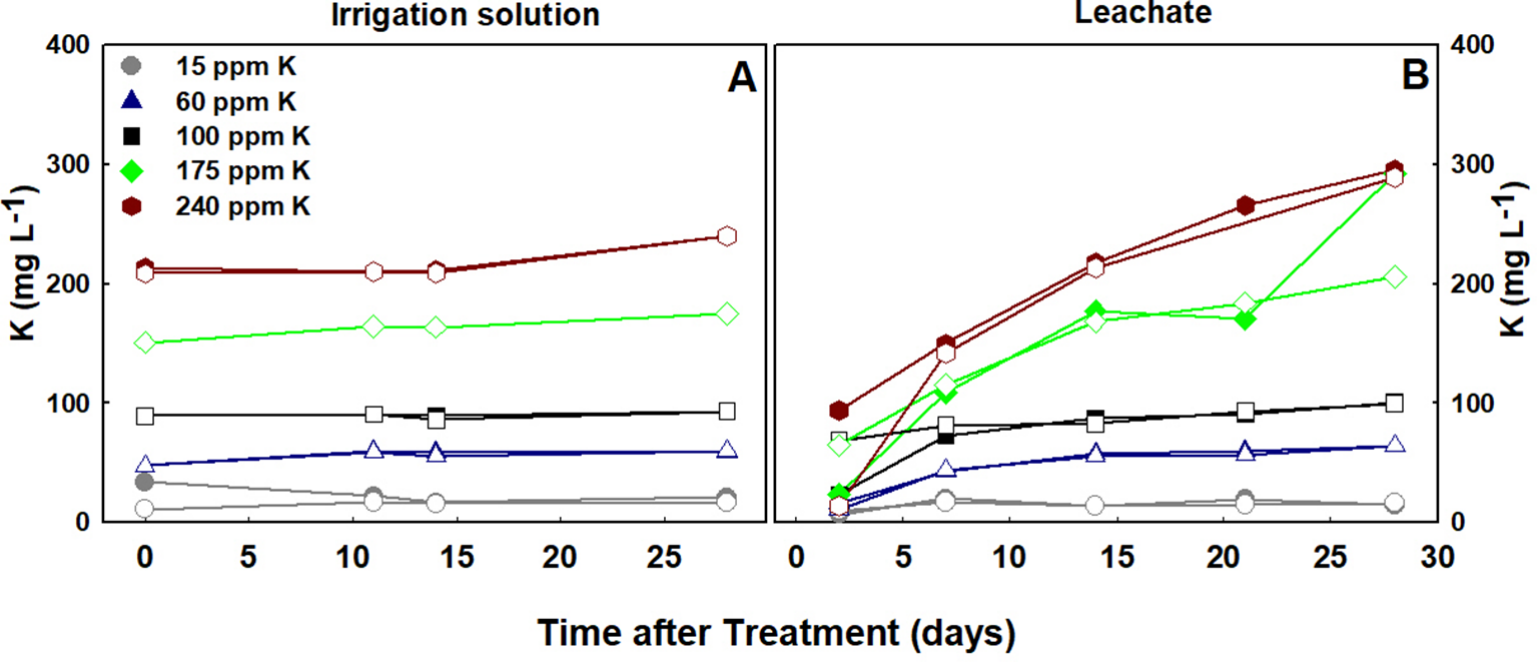
Concentrations of K in the irrigation solutions **(A)**, and leachates **(B)**, throughout the experiment duration. RM, filled symbol; DQ, empty symbol.

Routine chemical analyses of the fertigation (irrigation) solution demonstrated precise regulation of the treatment solutions. The concentration of K (**Figure 8A**) for both varieties was steady throughout the experiment duration in all five K treatments, and closely followed the designated treatments concentrations (**Figure 8A**). The concentration of K in the leachate solutions positively correlated with K supply in both varieties (**Figures 8B**). In the high K treatments, 175 and 240 ppm, the concentration in the leachate increased over time, suggesting over supply (**Figure 8B**).

### Plant Visual Characteristics

**Figure 9:**
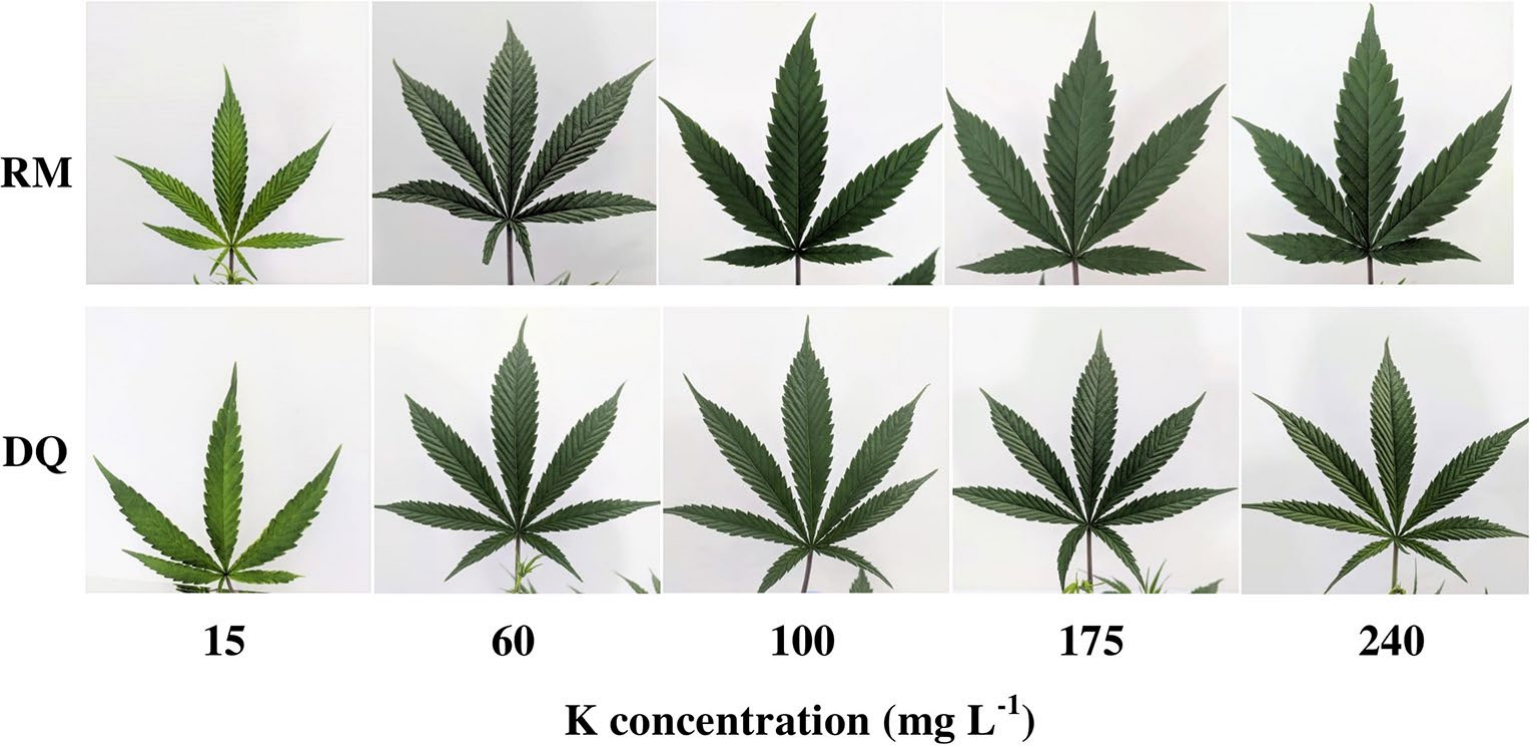
Visual appearance of leaves of two medical cannabis cultivars, RM (top row) and DQ (bottom row), which developed on plants receiving increasing K supply. From left to right: 15, 60, 100, 175, and 240 ppm K. Images of the youngest fully developed leaf on the main stem, taken 26 days after the initiation of the fertigation regime.

**Figure 10:**
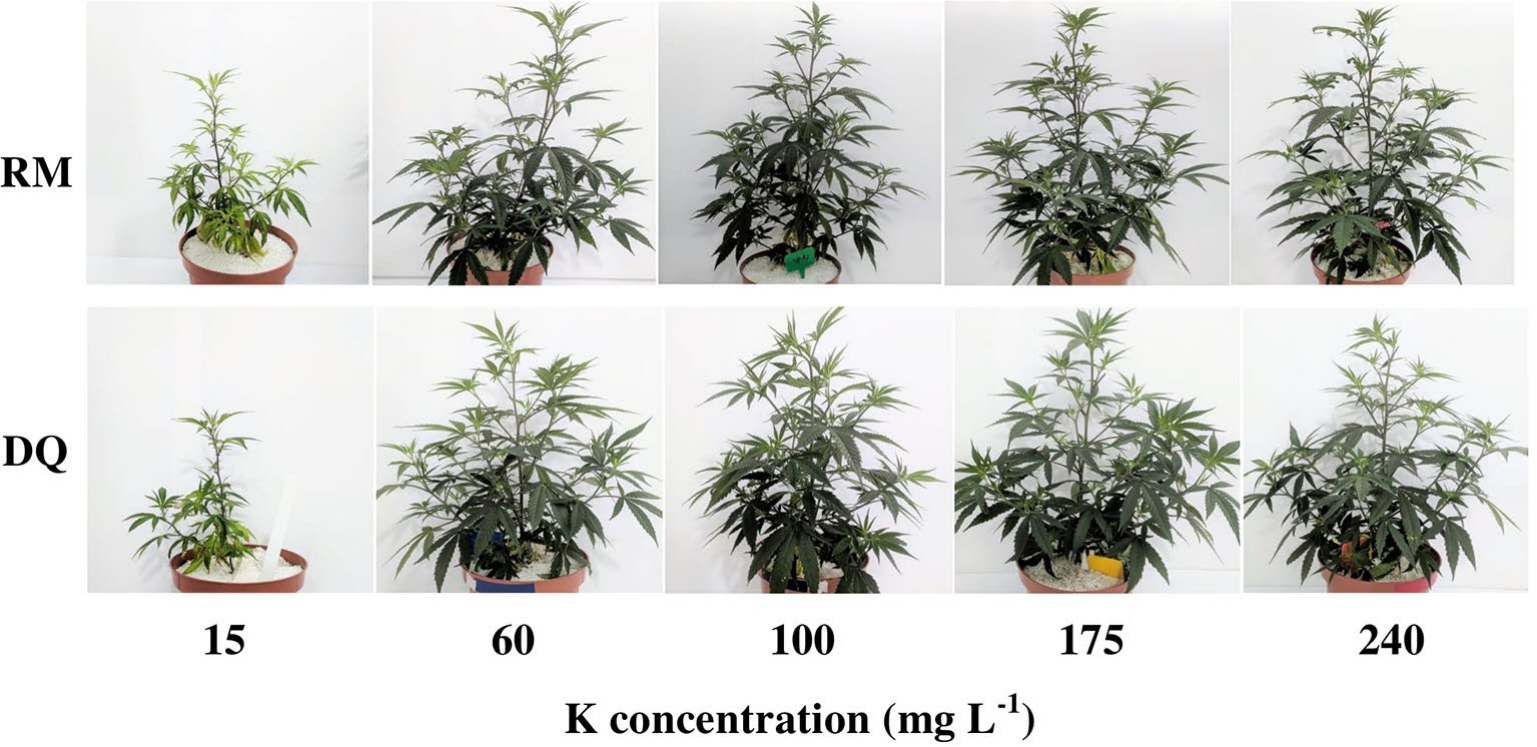
Plants of two medical cannabis cultivars, RM (top row) and DQ (bottom row), supplied with (from left to right): 15, 60, 100, 175, and 240 ppm K. Photographed 26 days after the initiation of the fertigation regime.

The visual appearance of the plants was affected similarly by the K treatments in the two cultivars, and reflected the morphological, chemical and physiological characteristics evaluated in the study (**Figures 9**–**10**). In the 15 ppm K treatment, the leaves were smaller, had fewer leaflets (in DQ), and showed advanced chlorosis typical of K deficiency (**Figure 9**). They were also developmentally inhibited, compared to the remaining K treatments that had larger leaves and darker green color (**Figure 9**). The chlorosis and restricted growth of the 15 ppm K treatment affected overall shoot growth, resulting in a smaller and thinner plants, compared to plants of all other treatments (**Figure 10**).

## Discussion

Mineral nutrition is one of the major factors affecting plant growth, development, and function. Optimal concentrations of mineral nutrients in the plant tissues and in the root solution vary for individual nutrients, and may differ between and within species. Furthermore, due to effects of specific nutrients on biochemical, physiological, and molecular processes, nutrition may need to be adjusted for directing a required metabolic process or a preferred developmental scheme, such as a vegetative or reproductive development. In the present study we report changes to the ionome and to plant development and function resulting from the intensity of K supply, comparatively for two cultivars of medical cannabis.

### Plant Growth and Development

Genetic variability within plant species results in genotypes with different developmental, physiological, and biochemical traits. Variability in response of roots and shoots of plant genotypes to growing conditions is well documented for numerous plant species (Antonio et al., 2019; Martins et al., 2016; Queiroz et al., 2019). For *C. sativa*, drug-type strains are known to vary in morphological and chemical characteristics, but responses to cultivation and environmental conditions are not known. In the present study we report that while the response to low, sub-optimal supply of K was similar for the genotypes studied, developmental differences between genotypes emerged under higher K concentration.

In both genotypes, biomass deposition was affected by K inputs but the response varied between the genotypes. In RM, growth positively responded to increase in K supply up to 175 ppm K (**Figures 1A–C**), as can be seen from the increase in leaves and root biomass (**Figures 1A, C**), stem diameter (**Figure 2E**), and internode elongation rate (**Figures 2E, G**), but decreased with further increase to 240 ppm K, rendering 175 ppm as the optimum concentration for this genotype. While DQ suffered as well from insufficient K supply under the 15 ppm K treatment, increasing K supply in the range of 60–175 ppm K did not affect plant development. Surprisingly, further increase in K supply, to the level of 240 ppm K, stimulated rather than restricted growth and development of this genotype.

The increase in biomass production with the increase in K at the lower concentration range in both genotypes represent mitigation of restricted supply, satisfying demands for facilitating optimal growth. The concentration range at which plant performance improves with increased supply is defined a “deficiency range,” which is well documented to vary between species (Marchner, 2012) and in some cases also between genotypes. The lower requirement of K supply for optimal development in RM may be the outcome of the smaller shoot and root morphology in this genotype, compared to the larger vegetative plant body of DQ. RM might have therefore been less prone to deficiency. Potassium plays an active part in the physiological regulation of crop processes (Wang and Wu, 2017), facilitating functions such as ion uptake and transport, protein synthesis, stomatal regulation, enzymatic activity, and regulation of gene expression. The observed growth stimulation with increased K supply at the low concentration range in the two cannabis genotypes can result from effects of the deficiency on individual factors or combination of mechanisms as was demonstrated for numerous plant species. In tomatoes for example, the suppression of stem expansion under K deficiency, which was apparent in both medical cannabis genotypes as well, was concluded to result from a reduction in water supply to the growing stem by K-deficiency induced reduction in aquaporins and K-channels activity (Fromm, 2010; Kanai et al., 2011). Similar to our results, addition of K led to development of thicker stems and higher shoot biomass.

Potassium is a major player in regulation of plant water relations (Mengel and Arneke, 1982), osmo-regulation (Lauchli and Pfluger, 1978), and stomatal opening (Fischer, 1968, Fischer, 1971; Outlaw, 1983) and hence plant development (Prajapati and Modi, 2012; Wang et al., 2013). The source of the different development response of the two medical cannabis genotypes to K supply, roots in the impact of K availability on the physiological status, mostly water relations, of the tissue. Although in the RM plants the rate of photosynthesis was not affected by the K treatments, slower transpiration rate with reduced stomatal conductance were induced by K-deficiency (15 ppm K) (**Figure 5**) resulting in restricted growth but higher water use efficiency. Environmental conditions are known to affect carbon fixation (Rodrigues et al., 2016). The change in water relations in the 15 ppm K treatment was apparent also from the lower osmotic potential and relative water content in the leaf tissue, and higher %DW, i.e., lower percentage of water in the tissue. The combined effects of K deficiency and altered water status caused tissue damage, as was apparent from the higher membrane leakage (**Figure 6E**), and resulted in restriction of development. The relative water content and osmotic potential were lower in the 15 ppm K treatment in DQ as well (**Figure 6**), but growth was restricted by a different mechanism. That is, in this genotype, transpiration rate and stomatal conductance were not impaired under the deficient K supply (15 ppm K), but net photosynthesis rate was reduced, resulting in higher CO_2_ concentration in the intercellular space, due to a decrease in the consumption of CO_2_ as a substrate for photosynthesis (**Figure 5**). Membrane leakage, i.e., tissue stress was not affected, but the tissue did suffer from water shortage, resulting in lower %DW in the evaporating tissues of the leaves. Despite the variation in net photosynthesis rate and gas exchange parameters between the genotypes, the values obtained are within the range obtained in former measures conducted for indoor grown medical cannabis (Chandra et al., 2011).

In both genotypes, K supply had a significant effect on the photosynthetic pigments in the tissue; positively correlating with chlorophyll a and carotenoids contents (**Figures 7A, C**). Moreover, chlorophyll b as well was affected by K supply but K demands for optimal accumulation were satisfied already under 60 ppm K inputs (**Figure 7B**). As K is not a constituent of these molecules, the impact it has on their biosynthesis is indirect, and we suggest that the decrease in concentration of the pigments under 15 ppm K results from inhibition of N availability in the leaf cells, N being a central constituent of these molecules (Taiz and Zeiger, 2010). Furthermore, K, as a cofactor, is involved in the activity of a large number of vital enzymes (Evans and Sorger, 1966; Prajapati and Modi, 2012), that affect also metabolism and catabolism of plant pigments. Reduced chlorophyll concentration induced leaf chlorosis, and the impaired water relations and reduced availability of K resulted in a morphological response with development of smaller leaves and leaflets and fewer leaflets.

Excess of K supply induced contrasting effects on the two genotypes. The difference in plant biomass between the genotypes in the 240 ppm K treatment results from differential effect of the high K supply on development of the side branches. In RM the length of the side branches was inhibited by 21% as K supply increased from 175 to 240 ppm, while in DQ the side branches length increased by 14% (data not shown). K effect on side branch development is known also for other plants (Madgwick, 2011) and is likely to have a large effect on total biomass accumulation even without an effect on branching. The elevated biomass deposition in DQ under high K supply is the outcome of an increase leaf biomass of the side branches as well as in stem diameter (data not shown), resulting in bushier bigger plants. K is known to affect stem diameter and fiber yield and quality in other plant species as well (Derrick et al., 2013).

Accumulation of nutrients above the optimal level required for plant growth and function i.e., “luxury consumption,” is a process described for numerous plant species, mostly related to K uptake (Bartholomew and Janssen, 1929). “Luxury consumption” of K usually does not affect growth and development of plants, but it was previously reported for numerous species including cotton, that excessive K fertilizer reduces plant biomass (Chen et al., 2017). In the case of the two medical cannabis genotypes, the mechanisms for the contrasting developmental response are not clear and require further study. Specifically, the higher concentration of K in stems of DQ compared to RM under 240 ppm K, (higher by 20%) is unlikely the cause of the observed growth stimulation in this genotype, because the increased in K concentration in the shoot under 60–175 ppm K supply did not affect plant development, categorizing the increased K uptake in this range as luxury consumption. In RM as well, changes in K concentrations in the shoot do not present a direct cause for growth restriction under 240 ppm K compared to lower supply rates since K concentrations were not affected considerably (**Figure 3**). No apparent changes in any other macro or micronutrients (**Figures 3**, **4**), carbon fixation and gas exchange parameters (**Figure 5**) or water relations parameters (**Figure 6**) were identified as potential causes for the developmental response and genotypic differences under 240 ppm K.

Interestingly, the distribution of K between plant organs differed for the two genotypes—while in RM concentrations in the roots and the shoot organs were similar, higher levels of K transport to the shoot were apparent in DQ. Consequently, supplied levels of K that restricted growth in RM, stimulated growth in DQ (**Figure 3A**). This suggests differential sensitivity of the cells from both genotypes to K, or more likely, involvement of a secondary-induced factor in the observed growth restriction. The variability in the response of the two genotypes to K supply, demonstrated by an optimum response curve in RM, and the three distinct response phases in DQ (**Figures 1A–C**), reveals as well genetic differences within the *C. sativa* species to mineral nutrition.

Nutrient use efficiency, e.g., the amount of biomass produced per K unit supplied, is a valued tool for evaluating the ability of the plant to utilize environmental inputs into yield or biomass production (Yasuor et al., 2013; Omondi et al., 2018). We report here for medical cannabis, a large decrease in KUE with the increase in K supply (**Figure 1D**). This points again at “luxury consumption” and suggests that, maximum efficiency is obtained under low K concentrations. For a high cash-crop like medical cannabis, it is likely that the marginal addition to the biomass at the vegetative stage, if proven to support better plant architecture for the reproductive phase, will be more significant than the fertilization expenses. Plant genotypes are known to vary also in nutrient use efficiency. In the present study KUE was higher in DQ compared to RM.

### Mineral Nutrients

The variability in the distribution patterns of the various macronutrient in the plant body result from uptake and translocation mechanisms. The increased concentration of K in the root solution increase overall cation concentration in the solution and hence competition for uptake of the positively charged cations. Reduction in Ca concentration in the plants under high K supply (**Figure 3E**), points at competition for uptake. Ca/K competition for root uptake (Johansen et al., 1968; Maas, 1969) and a resulting reduced Ca concentration in the shoot was documented for a variety of plant species (Fageria, 2001). Moreover, in-planta transport of these two ions is also competitive since high K concentrations decreased the amount of Ca arriving to the foliage, as already seen before (Overstreet et al., 1952; Bar-Tal and Pressman, 1996). Mg is another cation which was identified to compete with K for root uptake and translocation in the plant (Heenana and Campbell, 1981), but in cannabis K has a smaller influence on its translocation since Mg concentration began to decrease only in the 60 ppm K treatments (**Figure 3F**). The uptake and distribution of the two other major macronutrients, N and P, is not sensitive to K supply (**Figures 3B, C**), probably since their uptake into the root cells is as anions, in a mechanism less affected by cation concentrations and uptake (most of the N in the present study was supplied as NO_3_^-^) (White, 2012).

Concentration of K in the leachate solution is another indicator of plant requirement and uptake. In the present study, K concentration in the leachate was higher than in the irrigation solution only at the 175 and 240 ppm K treatments (**Figure 8B**), indicating that K supply under these treatments exceeded plant uptake. Nutrient concentration in the leachate is an integral result of water and mineral uptake by the plants. When water is taken up to a greater extent than a mineral, its concentration in the leachate will exceed the concentration in the irrigation solution. The concentration of K in the leachates of the three lower K treatments was similar to the concentration in the irrigation solution, demonstrating similar uptake rates of K and water. Under 175–240 ppm K application, the higher concentration of K in the leachate compared to the irrigation solution demonstrate that the rate of water uptake was higher than for K, resulting in an increase in K. This suggests that 175 ppm K is higher than the plant requirement.

Micronutrient uptake is a limiting growth factor for foliage and shoot development in many plant species under various growing conditions (Baszyński et al., 1978; Ohki et al., 1980; Clark, 1982; Webb and Loneragan, 1988; Yu and Rengel, 1999). No information is currently available about micronutrient requirements or effects on medical cannabis, and our results present initial understanding. Under the cultivation conditions and rate of nutrient supply at the present study, the two cannabis cultivars examined did not show any signs of micronutrient deficiencies, suggesting sufficient supply (**Figure 4**). Surprisingly, most micronutrients and the beneficial element Na, did not translocate to the shoot but tended to accumulate in the root. Zinc, Mn, Fe, Cu, and Cl as well as Na concentrations were all higher in the root compared to the shoot, suggesting a compartmentation strategy for temporary storage, or for prevention of access concentrations at the shoot tissues. Results of the comparative analyses point at competitive uptake between K and Mn, Zn and Fe, since concentrations of the latter decreased with increased K supply. Na uptake was less affected by this competition (**Figure 4E**), in accord with its known strong competition abilities with K for root uptake (Amtmann and Leigh, 2010), or due to its very low concentration in the fertigation solution, which was prepared with distilled water. The uptake of another micronutrient, Cl, was not affected by cultivar or K supply (**Figure 4F**), probably because its concentrations was low and within the range accepted as optimal to most plants (Parker et al., 1983; Marchner, 2012).

Some information for micronutrient uptake by *C. sativa* L. is available for industrial, fiber-type, Hemp. It is used for phytoremediation, due to its known ability to absorb heavy metals from the soil and tolerate high accumulation in its tissues (Linger et al., 2002; Ahmad et al., 2016). Contradictory to our expectation for similar high uptake rates of micronutrient cations into the medical cannabis plants, these Hemp properties were not found in the medical type varieties. The difference between the Hemp and medical cannabis response could result from several factors; 1. The distinct plant genetics of Hemp may express enhanced uptake mechanisms for heavy metals; 2. Differences in chemical and physical properties (such as pH, chelate diversion, and cation exchange capacity) between the rhizosphere of the soilless cultivated medical cannabis and the soil-grown Hemp (Landi, 1997; Aubin et al., 2015), may have affected root nutrient availability and uptake; 3. The growth period of the medical cannabis cultivars was short compared to a standard growth period of Hemp (Van der Werf and Van den Berg, 1995; Linger et al., 2002; Vera et al., 2010), resulting in overall lower amounts of metal accumulation in the medical cannabis cultivars.

## Author Contributions

NB planned the experiments. AS carried out the experiments. NB and AS wrote the manuscript. MS constructed the fertilizers compositions.

## Conflict of Interest

The authors declare that the research was conducted in the absence of any commercial or financial relationships that could be construed as a potential conflict of interest.

## References

[B1] Ahmad R. Tehsin Z. Malik S. T. Asad S. A. Shahzad M. Bilal M. (2016). Phytoremediation potential of hemp (Cannabis sativa L.): Identification and characterization of heavy metals responsive genes. Clean - Soil Air Water 44, 195–201. 10.1002/clen.201500117

[B2] Amtmann A. Leigh R. (2010). “Ion homeostasis,” in Abiotic stress adaptation in plants: molecular and genomic foundation. Eds. Pareek A. Sopory S. K. Bohnert H. J. (Dordrecht, The Netherlands: Springer), 245–262. 10.1007/978-90-481-3112-9

[B3] Antonio J. Giles D. Duim A. Luiz F. Mitsuko E. Cochicho J. (2019). Divergence and genetic parameters between coffea sp. genotypes based in foliar morpho-anatomical traits. Sci. Hortic. (Amsterdam) 245, 231–236. 10.1016/j.scienta.2018.09.038

[B4] Aubin M. Seguin P. Vanasse A. Tremblay G. F. Mustafa A. F. Charron J. (2015). Industrial hemp response to nitrogen, phosphorus, and potassium fertilization. Crop. Forage Turfgrass Manag. 1, 1–10. 10.2134/cftm2015.0159

[B5] Bar-Tal A. Pressman E. (1996). Root restriction and potassium and calcium solution concentrations affect dry-matter production, cation uptake, and blossom-end rot in greenhouse tomato. J. Am. Soc. Hort. Sci 121, 649–655. 10.21273/JASHS.121.4.649

[B6] Bartholomew R. P. Janssen G. (1929). Luxury consumption of potassium by plants and its significance. Agron. J. 21, 751–765. 10.2134/agronj1929.00021962002100070005x

[B7] Baszyński T. Ruszkowska M. Król M. Tukendorf A. Wolińska D. (1978). The effect of copper deficiency on the photosynthetic apparatus of higher plants. Z. für Pflanzenphysiol. 89, 207–216. 10.1016/S0044-328X(78)80012-9

[B8] Bernstein N. Gorelick J. Koch S. (2019a). Interplay between chemistry and morphology in medical cannabis (Cannabis sativa L.). Ind. Crops Prod. 129, 185–194. 10.1016/j.indcrop.2018.11.039

[B9] Bernstein N. Gorelick J. Zerahia R. Koch S. (2019b). Impact of N, P, K and humic acids supplementation on the chemical profile of medical cannabis (Cannabis sativa L). Front. Plant Sci. 10, 736. 10.3389/fpls.2019.00736 31263470 PMC6589925

[B10] Bernstein N. Ioffe M. Luria G. Bruner M. Nishri Y. Philosoph-Hadas S. (2011). Effects of K and N nutrition on function and production of Ranunculus asiaticus. Pedosphere 21, 288–301. 10.1016/S1002-0160(11)60129-X

[B11] Bernstein N. Shoresh M. Xu Y. Huang B. (2010). Free radical biology & medicine involvement of the plant antioxidative response in the differential growth sensitivity to salinity of leaves vs roots during cell development. Free Radic. Biol. Med. 49, 1161–1171. 10.1016/j.freeradbiomed.2010.06.032 20619339

[B12] Bócsa I. Máthé P. Hangyel L. (1997). Effect of nitrogen on tetrahydrocannabinol (THC) content in hemp (Cannabis sativa L.) leaves at different positions. J. Int. Hemp. Assoc. 4, 80–81.

[B13] Cascio M. G. Pertwee R. G. Marini P., and H. (2017). “The pharmacology and therapeutic potential of plant cannabinoids,” in Cannabis sativa L. - botany and biotechnology. Eds. Chandra S. Lata H. ElSohly M. A. (Cham: Springer International Publishing), 207–225. 10.1007/978-3-319-54564-6_9

[B14] Chandra S. Lata H. Khan I. A. ElSohly M. A. (2011). Photosynthetic response of Cannabis sativa L., an important medicinal plant, to elevated levels of CO2. Physiol. Mol. Biol. Plants 17, 291–295. 10.1007/s12298-011-0066-6 23573021 PMC3550578

[B15] Chen B. Chai Z. Sheng J. Zhang W. Jiang P. (2017). Effects of potassium fertilizer on the physiological mechanisms of cotton fiber quality. Pak. J. Bot. 49, 935–943.

[B16] Clark R. B. (1982). Iron deficiency in plants grown in the great plains of the U.S. J. Plant Nutr. 5, 251–268. 10.1080/01904168209362955

[B17] Clarke R. C. Merlin M. D. (2013). Cannabis: evolution and ethnobotany. Los Angeles: University of California Press.

[B18] Coffman C. B. Gentner W. A. (1975). Cannabinoid profile and elemental uptake of Cannabis sativa L. as influenced by soil characteristics 1. Agron. J. 67, 491–497. 10.2134/agronj1975.00021962006700040010x

[B19] Covre A. M. Partelli F. L. Gontijo I. (2015). Distribuição do sistema radicular de cafeeiro conilon irrigado e não irrigado. Pesqui. Agropecuária Bras. 50, 1006–1016. 10.1590/S0100-204X2015001100003

[B20] Derrick M. O. Dimitra A. L. Tyson B. R. (2013). Potassium and stress alleviation: Physiological functions and management of cotton. J. Plant Nutr. Soil Sci. 176, 331–343. 10.1002/jpln.201200414

[B21] Eliašová A. Repčák M. Pastírová A. (2004). Quantitative changes of secondary metabolites of Matricaria chamomilla by abiotic stress. Z. fur Naturforsch. - Sect. C J. Biosci. 59, 543–548. 10.1515/znc-2004-7-817 15813377

[B22] Evans H. J. Sorger G. J. (1966). Role of mineral elements with emphasis on the univalent cations. Annu. Rev. Plant Physiol. 17, 47–76. 10.1146/annurev.pp.17.060166.000403

[B23] Fageria V. D. (2001). Nutrient interactions in crop plants. J. Plant Nutr. 24, 1269–1290. 10.1081/PLN-100106981

[B24] Figueiredo A. C. Barroso J. G. Pedro L. G. Scheffer J. J. C. (2008). Factors affecting secondary metabolite production in plants: volatile components and essential oils. Flavour Fragr. J. 23, 213–226. 10.1002/ffj.1875

[B25] Finnan J. Burke B. (2013). Potassium fertilization of hemp (Cannabis sativa). Ind. Crop. Prod. 41, 419–422. 10.1016/j.indcrop.2012.04.055

[B26] Fischer R. A. (1968). Stomatal opening: role of potassium uptake by guard cells. Science (80-.). 160, 784–785. 10.1126/science.160.3829.784 5646418

[B27] Fischer R. A. (1971). Role of potassium in stomatal opening in the leaf of Vicia faba. Plant Physiol. 47, 555–558. 10.1104/pp.47.4.555 16657659 PMC396725

[B28] Fromm J. (2010). Wood formation of trees in relation to potassium and calcium nutrition. Tree Physiol. 30, 1140–1147. 10.1093/treephys/tpq024 20439254

[B29] Gomes W. R. Rodrigues W. P. Vieira H. D. Oliveira M. G. Dias J. R. M. (2016). Genetic diversity of standard leaf nutrients in Coffea canephora genotypes during phenological phases. Genet. Mol. Res. 15, 15048839. 10.4238/gmr.15048839 27808383

[B30] Gorelick J. Bernstein N. (2014). “Elicitation: an underutilized tool in the development of medicinal plants as a source of therapeutic secondary metabolites,” in Advances in agronomy (Amsterdam: Elsevier Inc). 201–230. 10.1016/B978-0-12-800138-7.00005-X

[B31] Greer G. R. Grob C. S. Halberstadt A. L. (2014). PTSD symptom reports of patients evaluated for the New Mexico medical cannabis program. J. Psychoact. Drugs 46, 73–77. 10.1080/02791072.2013.873843 24830188

[B32] Grzebisz W. Gransee A. Szczepaniak W. Diatta J. (2013). The effects of potassium fertilization on water-use efficiency in crop plants. J. Plant Nutr. Soil Sci. 176, 355–374. 10.1002/jpln.201200287

[B33] Haney A. Kutscheid B. B. (1973). Quantitative variation in the chemical constituents of marihuana from stands of naturalized Cannabis sativa L. @ in East-Central Illinois. Econ. Bot. 27, 193–203. 10.1007/BF02872989

[B34] Heenana D. P. Campbell L. C. (1981). Influence of potassium and manganese on growth and uptake of magnesium by soybeans (Glycine max (L.) Merr. cv. Bragg). Plant Soil 61, 447–456. 10.1007/BF02182025

[B35] Intrigliolo D. S. Castel J. R. (2005). Effects of regulated deficit irrigation on growth and yield of young Japanese plum trees. J. Hort. Sci. Biotechnol. 80, 177–182. 10.1080/14620316.2005.11511913

[B36] Ivonyi I. Izsoki Z. Van der Werf H. M. (1997). Influence of nitrogen supply and P and K levels of the soil on dry matter and nutrient accumulation of fiber hemp (Cannabis sativa L.). J. Int. Hemp. Assoc. 4, 84–89.

[B37] Johansen C. Edwards D. G. Loneragan J. F. (1968). Interactions between potassium and calcium in their absorption by intact barley plants. I. Effects of Potassium on Ca absorption. Plant Physiol. 43, 1717–1721. 10.1104/pp.43.10.1717 16656960 PMC1087064

[B38] Kanai S. Moghaieb R. E. El-Shemy H. A. Panigrahi R. Mohapatra P. K. Ito J. (2011). Potassium deficiency affects water status and photosynthetic rate of the vegetative sink in green house tomato prior to its effects on source activity. Plant Sci. 180, 368–374. 10.1016/j.plantsci.2010.10.011 21421382

[B39] Landi S. (1997). Mineral nutrition of Cannabis sativa L. J. Plant Nutr. 20, 311–326. 10.1080/01904169709365252

[B40] Lash R. (2010). Hemp: the crop for the seventh generation. Am. Indian Law Rev. 27, 313–356. 10.2307/20070692

[B41] Lauchli A. Pfluger R. (1978). “Potassium transport through plant cell membranes and metabolic role of potassium in plants,” in Potassium research — review and tren (Bern: International Potash Institute), 111–163.

[B42] Leizer C. Ribnicky D. Poulev A. Dushenkov S. Raskin I. (2000). The composition of hemp seed oil and its potential as an important source of nutrition. J. Nutraceuticals Funct. Med. Foods 2, 35–53. 10.1300/J133v02n04_04

[B43] Lichtenthaler H. K. Wellburn A. R. (1983). Determinations of total carotenoids and chlorophylls a and b of leaf extracts in different solvents. Biochem. Soc. Trans. 11, 591–592.

[B44] Linger P. Mussig J. Fisher H. Kobert J. (2002). Industrial hemp (Cannabis sativa L.) growing on heavy metal contaminated soil: fibre quality and phytoremediation potential. Ind. Crop. Prod. 16, 33–42. 10.1016/S0926-6690(02)00005-5

[B45] Lu S. Wang Z. Niu Y. Guo Z. Huang B. (2008). Antioxidant responses of radiation-induced dwarf mutants of bermudagrass to drought stress. J. Am. Soc. Hort. Sci. 133, 360–366. 10.21273/JASHS.133.3.360

[B46] Maas E. V. (1969). Calcium uptake by excised maize roots and interactions with alkali cations. Plant Physiol. 44, 985–989. 10.1104/pp.44.7.985 16657169 PMC396202

[B47] Madgwick H. A. I. (2011). Branch growth of Pinusresinosa Ait. with particular reference to potassium nutrition. Can. J. For. Res. 5, 509–514. 10.1139/x75-074

[B48] Malash N. Flowers T. J. Ragab R. (2005). Effect of irrigation systems and water management practices using saline and non-saline water on tomato production. Agric. Water Manag. 78, 25–38. 10.1016/j.agwat.2005.04.016

[B49] Marchner H. (2012). Marschner’s mineral nutrition of higher plants. 3rd ed Ed. Marchner P. (London: Academic Press).

[B50] Martins M. Q. Rodrigues W. P. Fortunato A. S. Leitão A. E. Rodrigues A. P. Pais I. P. (2016). Protective response mechanisms to heat stress in interaction with high [CO2] conditions in Coffea spp. Front. Plant Sci. 7, 947. 10.3389/fpls.2016.00947 27446174 PMC4925694

[B51] Mengel K. Arneke W. W. (1982). Effect of potassium on the water potential, the pressure potential, the osmotic potential and cell elongation in leaves of Phaseolus vulgaris. Physiol. Plant. 54, 402–408. 10.1111/j.1399-3054.1982.tb00699.x

[B52] Naftali T. Bar-Lev Schleider L. Dotan I. Lansky E. P. Sklerovsky Benjaminov F. Konikoff F. M. (2013). Cannabis induces a clinical response in patients with crohn’s disease: A prospective placebo-controlled study. Clin. Gastroenterol. Hepatol. 11, 1276–1280. 10.1016/j.cgh.2013.04.034 23648372

[B53] Nascimento N. C. Fett-Neto A. G. (2010). “Plant secondary metabolism and challenges in modifying its operation: An overview,” in Plant secondary metabolism engineering. Methods in molecular biology (methods and protocols). Ed. Fett-Neto A. G. (Totowa, NJ: Humana Press), 1–13. 10.1002/9780470015902.a0001812.pub2 20552440

[B54] Ohki K. Wilson D. O. Anderson O. E. (1980). Manganese deficiency and toxicity sensitivities of soybean cultivars. Agron. J. 72, 713–716. 10.2134/agronj1980.00021962007200050005x

[B55] Omondi J. O. Lazarovitch N. Rachmilevitch S. Boahen S. Ntawuruhunga P. Sokolowski E. (2018). Nutrient use efficiency and harvest index of cassava decline as fertigation solution concentration increases. J. Plant Nutr. Soil Sci. 181, 644–654. 10.1002/jpln.201700455

[B56] Outlaw W. H. Jr. (1983). Current concepts on the role of potassium in stomatal movements. Physiol. Plant. 59, 302–311. 10.1111/j.1399-3054.1983.tb00775.x

[B57] Overstreet R. Jacobson L. Handley R. (1952). The effect of calcium on the absorption of potassium by barley roots. Plant Physiol. 27, 583–590. 10.1104/pp.27.3.583 16654480 PMC547961

[B58] Parker M. B. Gascho G. J. Gaines T. P. (1983). Chloride toxicity of soybeans grown on atlantic coast flatwoods soils. Agron. J. 75, 439–443. 10.2134/agronj1983.00021962007500030005x

[B59] Pettigrew W. T. Meredith W. R. (1997). Dry matter production, nutrient uptake, and growth of cotton as affected by potassium fertilization. J. Plant Nutr. 20, 531–548. 10.1080/01904169709365272

[B60] Prajapati K. Modi H. A. (2012). The importance of potassium in plant growth - A review. Indian J. Plant Sci. 1, 177–186.

[B61] Queiroz M. Luiz F. Golynski A. De Sousa Pimentel N. Ferreira A. De Oliveira Bernardes C. (2019). Adaptability and stability of Coffea canephora genotypes cultivated at high altitude and subjected to low temperature during the winter. Sci. Hortic. (Amsterdam) 252, 238–242. 10.1016/j.scienta.2019.03.044

[B62] Rodrigues W. P. Martins M. Q. Fortunato A. S. Rodrigues A. P. Semedo J. N. Simões-Costa M. C. (2016). Long-term elevated air [CO2] strengthens photosynthetic functioning and mitigates the impact of supra-optimal temperatures in tropical Coffea arabica and C. canephora species. Global Change Biol. 22, 415–431. 10.1111/gcb.13088 26363182

[B63] Shoresh M. Spivak M. Bernstein N. (2011). Free radical biology & medicine involvement of calcium-mediated effects on ROS metabolism in the regulation of growth improvement under salinity. Free Radic. Biol. Med. 51, 1221–1234. 10.1016/j.freeradbiomed.2011.03.036 21466848

[B64] Small E. (2017). “Classification of Cannabis sativa L. @ in relation to agricultural, biotechnological, medical and recreational utilization,” in Cannabis sativa L. - Botany and biotechnology. Eds. Chandra S. Lata H. ElSohly M. A. (Cham, Switzerland: Springer International Publishing), 1–62. 10.1007/978-3-319-54564-6_1

[B65] Szczerba M. W. Britto D. T. Kronzucker H. J. (2009). K+ transport in plants: physiology and molecular biology. J. Plant Physiol. 166, 447–466. 10.1016/j.jplph.2008.12.009 19217185

[B66] Taiz L Zeiger E, editors. (2010). “Photosynthesis: The light reactions,” in Plant physiology (Sunderland, MA: Sinauer Associates), 163–198.

[B67] Tsialtas I. T. Shabala S. Baxevanos D. Matsi T. (2016). Effect of potassium fertilization on leaf physiology, fiber yield and quality in cotton (Gossypium hirsutum L.) under irrigated Mediterranean conditions. F. Crop. Res. 193, 94–103. 10.1016/j.fcr.2016.03.010

[B68] Usherwood N. R. (1985). “The role of potassium in crop quality,” in Potassium in agriculture. Ed. Munson R. (Madison, Wisconsin: ASA, CSSA and SSSA), 489–513. 10.2134/1985.potassium.c21

[B69] Van der Werf H. M. G. Van den Berg W. (1995). Nitrogen fertilization and sex expression affect size variability of fibre hemp (Cannabis sativa L.). Oecologia 103, 462–470. 10.1007/BF00328684 28306994

[B70] Vera C. L. Malhi S. S. Phelps S. M. May W. E. Johnson E. N. (2010). N, P, and S fertilization effects on industrial hemp in Saskatchewan. Can. J. Plant Sci. 90, 179–184. 10.4141/CJPS09101

[B71] Wang H. Wu L. Cheng M. Fan J. Zhang F. Zou Y. (2018). Coupling effects of water and fertilizer on yield, water and fertilizer use efficiency of drip-fertigated cotton in northern Xinjiang, China. F. Crop. Res. 219, 169–179. 10.1016/j.fcr.2018.02.002

[B72] Wang M. Zheng Q. Shen Q. Guo S. (2013). The critical role of potassium in plant stress response. Int. J. Mol. Sci. 14, 7370–7390. 10.3390/ijms14047370 23549270 PMC3645691

[B73] Wang Y. Wu W. H. (2017). Regulation of potassium transport and signaling in plants. Curr. Opin. Plant Biol. 39, 123–128. 10.1016/j.pbi.2017.06.006 28710919

[B74] Webb M. J. Loneragan J. F. (1988). Effect of zinc deficiency on growth, phosphorus concentration, and phosphorus toxicity of wheat plants. Soil Sci. Soc. Am. J. 52, 1676–1680. 10.2136/sssaj1988.03615995005200060032x

[B75] White P. J. (2012). “Ion uptake mechanisms of individual cells and roots: short-distance transport,” in Marschner’s mineral nutrition of higher plants. Ed. Marchner P. (London: Academic Press), 7–47. 10.1016/B978-0-12-384905-2.00002-9

[B76] Yasuor H. Ben-Gal A. Yermiyahu U. Beit-Yannai E. Cohen S. (2013). Nitrogen management of greenhouse pepper production: agronomic, nutritional, and environmental implications. HortScience 48, 1241–1249. 10.21273/HORTSCI.48.10.1241

[B77] Yu Q. Rengel Z. (1999). Micronutrient deficiency influences plant growth and activities of superoxide dismutases in narrow-leafed lupins. Ann. Bot. 83, 175–182. 10.1006/anbo.1998.0811

[B78] Zuardi A. W. (2006). History of cannabis as a medicine: a review. Rev. Bras. Psiquiatr. 28, 153–157. 10.1590/S1516-44462006000200015 16810401

